# A qualitative study of differences in palliative care in hospitals and traditional home-based care in Cameroon

**DOI:** 10.1186/s12904-025-01868-2

**Published:** 2025-10-06

**Authors:** Emmanuel Aoudi Chance, Papa Théophile, Louise Renee Loe

**Affiliations:** 1 VID Specialized University, Ulriksdal 10, Bergen, Norway; 2 EELC, Hôpital Protestant de Ngaoundéré, BP 06, Ngaoundéré, Cameroon; 3 PUCA, Rue 1, 161 Rue Djoungolo, BP 4011, Yaoundé, Cameroon

**Keywords:** Palliative care, Hospital-based care, Home-based care, Cultural adaptation, Cameroon healthcare, Care quality

## Abstract

**Background:**

Cameroon’s palliative care system employs both hospital-based and traditional home-based methods, each influenced by cultural, structural, and practical considerations. This study investigated nurses’ and family caregivers’ perceptions of the differences between the two care settings, focusing on quality of care, cultural adaptation, and patient dignity.

**Methods:**

This qualitative study used semi-structured interviews to explore palliative care experiences in hospital and home-based settings in Cameroon. Participants were selected using a purposeful sampling strategy to gather a diverse range of experiences. Thematic analysis was used to identify key insights and recurring patterns related to the research questions.

**Results:**

Our findings highlight substantial differences between the provision of palliative care in hospitals and at home. While hospitals offer sophisticated medical interventions and standardized protocols, they face challenges due to resource limitations and conflicting cultural values with patient preferences. Home-based care, conversely, emphasizes personalized and culturally appropriate practices; however, it is often hindered by a lack of professional monitoring and adequate resources. Navigating these settings presents logistical and emotional difficulties for nurses, while family caregivers stress the significance of cultural factors in their caregiving decisions.

**Conclusion:**

A blended approach to palliative care, integrating the advantages of both hospital and home settings while mitigating their drawbacks, is crucial. In Cameroon, effective palliative care depends on policymakers and healthcare providers implementing culturally sensitive strategies and optimizing resource utilization.

## Introduction

According to the World Health Organization [54], palliative care aims to enhance the quality of life for both individuals facing life-threatening illnesses and their families by addressing their physical, psychological, social, and spiritual needs while upholding their dignity and alleviating suffering [54]. The rise in the global burden of chronic and terminal diseases, particularly in low- and middle-income countries (LMICs), underscores the urgency of prioritizing effective palliative care (Heller 23]. This study focused on Cameroon, where the need for improved palliative care is critical. This is because sub-Saharan Africa faces a particularly acute necessity for better palliative care due to high rates of conditions such as cancer, HIV/AIDS, and other non-communicable diseases, coupled with limited access to specialized medical services [40]. In Cameroon, despite this pressing need, palliative care remains underdeveloped due to systemic challenges that include a shortage of trained personnel, inadequate infrastructure, and the absence of robust palliative care policies [12].

Currently, palliative care services in Cameroon are largely concentrated in urban hospitals, which are often overstretched and lack specialized palliative care units [45]. While hospitals offer access to advanced medical treatments, their institutional nature can conflict with local cultural norms that emphasize personal connection, communal support, and the spiritual significance of dying at home [18]. Conversely, traditional home-based care is deeply rooted in Cameroonian culture, with families and communities typically assuming primary caregiving responsibilities in familiar surroundings that align with values that cherish dignity, family unity, and ancestral connection. However, this model often lacks adequate symptom management, clinical oversight, and caregiver training, thus potentially compromising the quality of care provided [50].

This contrast between hospital-based and home-based care highlights fundamental tensions between biomedical and cultural approaches to end-of-life care [29]. Hospitals prioritize clinical expertise, whereas home care reflects cultural values and social structures. Therefore, the intersection of culture, care delivery, and setting offers a crucial lens through which palliative care in Cameroon can be examined, as understanding their interplay can reveal opportunities for integrated models that balance medical effectiveness with cultural appropriateness.

Existing literature on palliative care in sub-Saharan Africa often focuses on medical interventions and resource availability, frequently overlooking the lived experiences of those directly involved in care delivery, such as nurses and family caregivers [ 39]. Furthermore, the influence of cultural norms on palliative care decisions and practices in Cameroon requires more in-depth investigation. While home-based care reflects traditional values, its limited integration with formal healthcare systems raises questions about how these paradigms can be effectively bridged to ensure high-quality, culturally sensitive care [28].

Therefore, this qualitative study addressed these gaps by exploring the perceptions of nurses and family caregivers in Cameroon regarding hospital-based and traditional home-based palliative care. By analyzing their experiences, this research aimed to inform the development of integrated care models that merge clinical competence with cultural sensitivity, tailored to the needs of diverse families and communities in resource-constrained and culturally rich environments. Understanding their perspectives is crucial for developing context-specific strategies to improve palliative care in Cameroon, thus potentially contributing to a broader understanding of the intersection of medical practices, cultural norms, and caregiving roles in similar settings.

### Research questions

#### Main research question

- How do nurses and family caregivers in Cameroon perceive the distinctions between hospital and home-based palliative care concerning quality, cultural relevance, and patient dignity?

#### Additional sub-questions

- What are the contrasting difficulties nurses encounter while delivering palliative care in Cameroonian hospitals as opposed to homes?
- What is the impact of Cameroonian cultural norms and traditions on palliative care choices and the experiences of patients?

## Methods

This study employed a qualitative descriptive design, enhanced using an interpretive lens, to capture rich, contextualized understandings of participants’ lived experiences [22]. The interpretive component acknowledges that cultural and social contexts profoundly shape how individuals perceive and articulate their experiences [35].

### Research design and rationale

The qualitative descriptive approach provided a detailed, accessible summary of caregiver experiences, while the interpretive lens supported deeper exploration of how cultural norms influence perceptions and care practices. This dual-layered design was particularly appropriate given the deeply rooted traditions, language diversity, and spiritual dimensions embedded in Cameroonian caregiving. By combining these methods, the study was better positioned to reveal both observable realities and underlying meanings within participants’ narratives.

### Sampling strategy

Purposive sampling was used to ensure the inclusion of participants with firsthand palliative care experience. Maximum variation sampling guided participant selection, ensuring diverse representation across regions, languages, religious affiliations, and caregiving roles. In total, 35 participants were recruited, comprising 15 registered nurses with at least one year of hospital-based palliative care experience and 20 family caregivers who had provided care in home settings within the past two years.

Recruitment was conducted via outreach presentations, flyer distribution, and collaboration with local health and religious institutions, considering Cameroon’s geographic and cultural diversity. Sampling continued until data saturation was achieved—that is, when no new themes emerged in successive interviews.

### Data saturation and information power

Data collection continued until thematic saturation was achieved, meaning no new conceptual categories or themes emerged from the interviews. We maintained detailed field notes and transcribed interviews concurrently with data collection, enabling iterative analysis and the identification of emerging themes. Regular discussions among the research team ensured that we were consistently assessing the richness and depth of the data. The 33rd interview marked a significant point in our data collection process, as we observed a notable decline in new information. By the 35th interview, the core themes and sub-themes had been thoroughly explored and became repetitive, indicating that thematic saturation had been reached.

In determining our sample size, we also considered the concept of “information power,” which emphasizes that the amount of information power a sample holds is related to the study’s aim, sample specificity, theoretical framework, quality of dialogue, and analysis strategy. Given our focused aim of exploring differences in palliative care, the relatively specific participant groups (patients, caregivers, and healthcare providers), and our in-depth interview approach, a sample size of 35 provided sufficient information power to generate meaningful insights and achieve our research objectives. The rich and detailed narratives collected from these diverse participants allowed for a comprehensive understanding of the nuances of palliative care experiences in both hospital and home-based settings in Cameroon.

### Inclusion and exclusion criteria

#### Inclusion criteria

- Registered nurses with ≥ one year of experience in hospital-based palliative care in Cameroon.
- Family caregivers who had provided palliative care at home within the past two years.
- Fluency in English, French, or a local language.

#### Exclusion criteria

- Individuals unable to consent or participate due to health or time constraints.

### Data collection procedures

Data were collected through in-person, semi-structured interviews held over one month at participants’ preferred locations, including hospitals, homes, and neutral community spaces. Interviews lasted 20–30 min, averaging at 25 min. This duration was chosen to respect participants’ emotional and logistical constraints, particularly due to the sensitive nature of palliative care and their often-limited availability.

Interviews were conducted by a trained multilingual team in English, French, or local languages. When language translation was required, bilingual translators (fluent in one local language and either English or French) were involved. A process of back-translation was employed on a sample of transcripts to verify the accuracy and cultural fidelity of the translations. All interviewers were trained to ensure consistency, neutrality, and cultural sensitivity.

To ensure comprehension and relevance for all participants, including family caregivers who might be less familiar with formal palliative care terminology, interviewers provided a brief, accessible explanation of palliative care as ‘care focused on providing relief from the symptoms and stress of a serious illness, aiming to improve quality of life for both the patient and the family,’ prior to the interview. The core interview guide remained consistent, but interviewers were also trained to rephrase questions and offer examples relevant to each participant’s specific caregiving context (hospital-based for nurses and home-based for family caregivers). This inclusive approach ensured that questions were understood and responses were rich and pertinent.

An interview guide was developed based on the literature review and refined through a pilot test. It included open-ended questions covering the following:

- Personal background in caregiving (hospital/home).
- Cultural influences on caregiving approaches.
- Challenges and emotional responses in caregiving.
- Experiences of support or lack thereof.
- Observed gaps in palliative care delivery.

The sample questions included were as follows:

- “Can you describe a situation where your cultural or spiritual beliefs influenced how you cared for your patient or loved one?”
- “What are the main difficulties you encounter when providing palliative care?”
- “How do hospital systems or home environments support—or hinder—your caregiving?”
- “Are there particular rituals or practices that are important to the person receiving care?”

### Interviewer training and reflexivity

Interviewers received comprehensive training that emphasized active listening to build rapport and trust, probing techniques to elicit more profound and more reflective responses, and a neutral stance to minimize interviewer bias. This training also included a strong focus on research ethics, ensuring participant protection and data integrity.

Reflexivity was a cornerstone of our research process. Each member of the research team, with their diverse experiences in healthcare, including direct involvement in palliative care settings in Cameroon, kept reflexive journals. These journals were used to critically examine how our backgrounds, beliefs, and positions influenced the data collection and interpretation processes. This ‘insider’ experience provided a nuanced understanding of the local context, cultural sensitivities, and existing healthcare challenges, which was invaluable during participant recruitment and the initial stages of data collection. It facilitated the establishment of rapport with participants and enabled a deeper understanding of their narratives.

To mitigate the potential for bias inherent in insider perspectives, several strategies were implemented to ensure rigor and reflexivity throughout the study. All interviewers underwent comprehensive training in qualitative research methodologies, including techniques for active listening, open-ended questioning, and bracketing personal assumptions. Regular debriefing sessions were conducted among the research team to discuss emerging themes, challenge interpretations, and critically reflect on potential biases. A dedicated researcher, not directly involved in palliative care provision, played a crucial role in data analysis, offering an external perspective to validate interpretations and ensure the objectivity of our findings.

### Data analysis

Data were analyzed thematically using Braun and Clarke’s [14] six-step framework, ensuring a rigorous and transparent process [14]:

1. Familiarization: Researchers read transcripts multiple times to immerse themselves in the data.
2. Initial coding: Transcripts were coded line-by-line to identify key segments of meaning.
3. Collaborative coding: Codes were compared and discussed by the research team to enhance reliability.
4. Theme development: Codes were grouped into themes reflecting recurring patterns across participants.
5. Review and refinement: Themes were checked for coherence and distinction, revisited against raw data.
6. Defining and naming themes: Final themes were defined using culturally appropriate language and grounded in participant narratives.

Then, the NVivo 14 software was used to organize, code, and retrieve data, enhancing transparency and traceability during the theme development. An example of theme development is a family caregiver stating, “At home, we can pray together and perform the rituals. It makes my father feel respected and calm, but I struggle to manage his pain properly.” This contributed to the “Cultural Alignment vs. Medical Expertise” theme, with sub-codes such as:

- “Cultural rituals enhance emotional well-being”.
- “Difficulty managing symptoms at home”.

Moreover, the theme of “Systemic Silence and Isolation” was informed by a nurse’s account: “Sometimes, we are the only ones talking to the family about death. There is no policy or support. It feels like we’re alone in it.” This theme captured feelings of institutional neglect and emotional burden among hospital-based caregivers. The themes were finalized through consensus in team meetings, ensuring robustness through researcher triangulation.

### Trustworthiness

To enhance the study’s trustworthiness, several methodological strategies were employed. To start with, credibility was ensured through member checking, where thematic summaries were shared with participants in their preferred language, allowing participants to confirm or clarify the researchers’ interpretations, with most affirming the themes’ accuracy and minor adjustments incorporated as needed. To support transferability, the study provided detailed descriptions of cultural contexts, caregiving environments, and participant backgrounds, enabling readers to assess the relevance of findings beyond the immediate sample. Further, dependability was strengthened by maintaining a transparent audit trail, including documentation of coding decisions, interviewer reflections, and peer debriefing memos. Finally, confirmability was supported through reflexive journaling and regular peer debriefings with experienced qualitative researchers not involved in the data collection. These practices helped minimize researcher bias and ensured a more balanced interpretation of the data.

### Ethical considerations

The study followed rigorous ethical standards, with all participants providing written informed consent after receiving verbal and written explanations of the study, including their right to withdraw at any time without penalty. Special attention was paid to cultural sensitivity in the consent process. When appropriate, verbal explanations were provided in local languages, and participants were invited to discuss their decision with a trusted family member or community leader, reflecting Cameroonian norms around collective decision-making.

Confidentiality was ensured through anonymized transcripts, coded identifiers, and secure storage of data. Recognizing the emotional sensitivity of palliative care, interviewers received additional training in trauma-informed communication, and participants showing signs of distress were offered support referrals. These included access to hospital-based counselors, chaplaincy services, or local religious leaders, depending on participant preference. Ethical approval was obtained from the Eglise Evangelique Lutherienne du Cameroun (EELC) and Hospital Protestant de Ngaoundere (Ref. No: 030/EELC/OSEELC/HPNDERE/DIR/24), aligning the study with both institutional and cultural ethical expectations.

## Results

### Participant demographics

A total of 35 semi-structured interviews were conducted for this study, comprising 15 nurses and 20 family caregivers across various hospital and home-based settings. Participants were selected to represent a diverse range of experiences in palliative care within the Cameroonian context. Interviews lasted 20–30 min, averaging at 25 min. The demographic characteristics are detailed in Table 2.

**Table 2:** Demographic characteristics of study participants

Characteristic	Nurses (*n* = 15)	Family caregivers (*n* = 20)
Gender	5 Male, 10 Female	8 Male, 12 Female
Age range	25–55 years	23–60 years
Years of experience	2–25 years	N/A
Setting	Hospital (15)	Home Care (20)
Relationship to patient	N/A	Spouse (13), Child (7)

Table 2 provides a demographic overview of the study participants, detailing the characteristics of both the nurses and family caregivers. The gender distribution shows that among the nurses, 5 were male and 10 were female, while among family caregivers, 8 were male and 12 were female. The age range for nurses was 25 to 55 years, and for family caregivers, it was 23 to 60 years. The professional experience of the nurses varied widely, with at least one year. Regarding their care settings, the nurses were from two distinct hospitals, whereas all the family caregivers were involved in home-based care. Table 2 also specifies the relationship of the family caregivers to the patients, with participants being either a spouse or a child.

### Thematic findings

This study identified six central themes that illuminate the lived experiences of nurses and family caregivers engaged in hospital- and home-based palliative care in Cameroon: (1) perceptions of care quality, (2) cultural adaptation, (3) challenges faced by nurses, (4) impact of cultural norms and traditions, (5) patient dignity, and (6) hybrid care models.

### Perceptions of care quality

Nurses and family caregivers held a nuanced view of care quality in both settings. Hospital-based care was acknowledged for providing easy access to medical resources and expertise in managing complex symptoms. As one nurse stated, *“The hospital has the equipment and medication to manage pain.”* This access was considered a key advantage for addressing critical medical needs effectively.

However, participants consistently highlighted significant obstacles to delivering optimal care in hospitals. Overcrowding and insufficient palliative care units, coupled with a shortage of adequately trained staff, were reported as major impediments to providing personalized care and emotional support. Pointing to the time pressure, a nurse noted, *“It often feels like we don’t have enough time to really connect with the patient or their family.”* Family caregivers echoed this sentiment, expressing feelings of isolation due to restricted visiting hours and limited involvement in care. One caregiver shared, *“In the hospital … it was hard for us to stay with him as much as we wanted. […] It felt very isolating.”* Thus, while hospitals offered medical expertise, their capacity to address the emotional and relational needs of patients and families was often compromised by systemic limitations.

Conversely, the perceived value of home care lay in its provision of emotional support, familiarity, and cultural appropriateness. A family caregiver explained, *“Taking care of my mother at home allowed her to be surrounded by family.”* This environment was seen as crucial for emotional well-being and preserving dignity.

Despite these benefits, significant concerns regarding home-based care were a lack of formal training among caregivers and limited access to pain management and other essential medical resources. A family caregiver stated, *“It was very hard to manage her pain without proper training or access to medication.”* The nurses corroborated this, pointing out that *“families often struggle without professional guidance*,* […] which affects the overall quality of care.”* Consequently, while home care offered a supportive and culturally resonant environment, its effectiveness was often undermined by a lack of medical expertise and resources.

In summary, participants perceived care quality as uneven. On one hand, hospital care offered medical advantages but often lacked the emotional and cultural sensitivity desired. On the other hand, home care provided emotional comfort and cultural relevance but was frequently hampered by inadequate training and medical resources. This duality underscores the need to bridge the strengths of both models to enhance palliative care in Cameroon.

### Cultural adaptation

This study suggests that hospital settings may not always align with cultural traditions that value family participation and comfortable, familiar surroundings when someone is nearing the end of their life. The nurses highlighted a conflict between hospital protocols, designed for efficiency and medical priorities, and certain cultural practices. For instance, one nurse mentioned, *“Hospitals don’t always accommodate our traditions*,* like prayer rituals or having extended family close by*,* which […] can make patients and families uncomfortable.”* Moreover, a common complaint is that hospitals’ inflexible systems do not allow for the cultural sensitivity needed in patient care. Although hospital staff were kind, family caregivers complained about feeling unsupported because their cultural needs were not understood. A family caregiver elaborated, *“The hospital staff was kind*,* […] but they didn’t understand why we needed to perform certain cultural ceremonies before my uncle passed away.”*

These statements reveal a considerable disparity between the hospital’s palliative care approach and Cameroonian cultural practices, particularly regarding the role of the family and the importance of a comfortable, home-like setting during end-of-life care. Despite having ample medical resources, hospitals frequently lack accommodation for the significant cultural practices of patients’ families.

The lack of cultural awareness in hospitals may cause distress for patients and their families, diminishing the quality of their care. This study demonstrates how challenging it is for families when hospital care does not respect their cultural values and traditions, emphasizing the importance of greater cultural sensitivity and flexibility in healthcare.

Participants stressed that home-based care better accommodates cultural values than hospital care, respecting traditions and enabling families to uphold their moral responsibilities. Strong cultural expectations often mean family members care for loved ones at home, emphasizing familiar surroundings and family support. According to a family caregiver, *“Caring for my father at home felt right because it aligns with our values*,* but it was overwhelming without external support.”* Similarly, according to a nurse, *“Home care respects cultural expectations*,* but families often face challenges when trying to balance tradition with the demands of caregiving.”*

These statements highlight the cultural importance of home-based care, allowing patients to be with loved ones and promoting dignity and comfort. However, although there are cultural advantages to home-based care, the constant burden on family caregivers, predominantly women, points to a critical need for increased support from community and institutional sources. Moreover, the lack of support left caregivers struggling to cope with the overwhelming demands of managing complex symptoms and providing constant care. In the words of one family caregiver, *“It was overwhelming without external support.”*

Caregiving places a heavy emotional, physical, and logistical burden on families, particularly those without professional support. Nurses also highlighted the conflict families face when balancing cultural expectations and the practicalities of care. One nurse commented, *“Home care respects cultural expectations*,* but families often face challenges when trying to balance tradition with the demands of caregiving.”* The complexities of home-based care highlight how cultural values, while offering comfort, can also create challenges when caregivers lack sufficient support.

While home-based end-of-life care is culturally appropriate, it underscores the need for greater support systems—from community or institutional sources—to ease the strain on family caregivers. Participants described caregiving as deeply embedded in cultural rituals such as communal prayers, ancestral blessings, and traditional medicine. Whereas family caregivers embraced these practices, nurses struggled to adapt biomedical routines to these cultural needs. As per a nurse, *“In the hospital*,* […] We cannot always allow family members to perform rituals. It creates tension*,* but we must follow procedures.”*

Thus, there was a shared call for hospital systems to better accommodate cultural expressions of care without compromising patient safety.

### Challenges faced by nurses

In hospital settings, nurses grapple with myriad challenges, including heavy workloads, limited resources, and the emotional strain of caring for the terminally ill. The lack of adequate palliative care support only exacerbates these difficulties. Furthermore, a dearth of training in culturally sensitive practices can impede effective care delivery.

A nurse succinctly captured this struggle by stating, *“We […] are understaffed*,* and there is little time to provide the emotional support that patients and families need in end-of-life care.”* Meanwhile, another nurse noted, *“Sometimes*,* we have the medical tools but lack […] the training in how to address patients’ cultural or spiritual needs.”*

These responses showed that nurses face significant challenges, especially when providing palliative care in hospitals. Overburdened by their work, nurses explained that they often lack the time to provide the emotional support patients and families require during end-of-life care. In the words of one nurse, *“…little time to provide the emotional support that patients…”* This response underscores the emotional toll on nurses, who must balance the technical and emotional aspects of care, a particularly crucial aspect of palliative care.

Nurses also cited the significant issue of the lack of alignment between medical resources and cultural competency. Although hospitals have medical resources to treat physical ailments, they often lack the training to address patients’ equally important cultural and spiritual needs for holistic care. A nurse commented, *“… lack the training in how to address patients’ cultural or spiritual needs.”*

This statement underscores the complex task of balancing medical proficiency with culturally sensitive palliative care, a crucial aspect of holistic care.

While hospitals boast ample medical resources, palliative care is marred by structural and training deficiencies. These shortcomings have a detrimental impact on patients’ emotional, cultural, and spiritual well-being; consequently, nurses advocated for a more holistic approach to hospital palliative care—one that encompasses adequate staff, resources, and cultural understanding. Logistical hurdles like accessing rural communities and working with informal caregivers can be problematic for home-based care nurses. Additionally, insufficient institutional support and a lack of collaboration with family caregivers may also affect them.

According to respondents, nurses providing home-based palliative care, particularly in rural settings, face significant logistical and relationship challenges. The nurses emphasized that reaching patients in these isolated locations presents a significant hurdle to delivering consistent care. *“Reaching rural areas is a big challenge. Sometimes*,* families call us*,* but the distance and lack of resources make it difficult to provide consistent care*,*”* said a nurse. *“In the home*,* we face resistance from families who rely on traditional remedies instead of following medical advice*,*”* stated another.

These statements stress how geographical and infrastructural barriers create significant delays and difficulties in providing timely and effective healthcare at home, especially in isolated communities.

Collaboration with families proved difficult for nurses, as families do not always comply with medical recommendations. Moreover, home healthcare often clashes with family preferences, as traditional practices sometimes outweigh medical advice, leading to the use of alternative remedies. The conflict between modern medical practices and established cultural values often challenges effective healthcare delivery.

The complexities of home healthcare are underscored by the challenges nurses face, which include logistical hurdles, navigating informal caregiving, and aligning medical recommendations with cultural norms. To ensure patients receive the best possible home care, it is imperative to foster greater institutional support and collaboration with family caregivers.

In this study, nurses reported moral distress, burnout, and lack of resources as major challenges in delivering palliative care. Notably, high workloads, poor infrastructure, and the burden of discharging patients into under-resourced home settings left many feeling helpless, with one nurse stating, *“Sometimes we send them home knowing they […] won’t get the pain relief they need. […] It’s heartbreaking.”*

Family caregivers also described the emotional toll of managing care alone, a situation worsened by inadequate access to pain medication and professional guidance.

### Impact of cultural norms and traditions

The preference for home-based care is often deeply rooted in cultural values, where families feel a moral obligation to care for their relatives. In this study, patterns showing potential conflicts between contemporary healthcare practices and traditional beliefs, such as the stigma surrounding illness and death or misconceptions about palliative care, may also emerge.

Participants stressed how strongly cultural norms influence the preference for home-based care and the difficulties this creates when traditional beliefs conflict with contemporary medical practices. The cultural expectation to provide in-home care for their loved ones is significant for family caregivers, but the burden becomes overwhelming when they lack the necessary skills.

According to a family caregiver, *“In our community*,* it is expected that family will care for the sick at home*,* but when we do not have the skills*,* it becomes very stressful.”*

This response underscores the conflict between cultural expectations and the practical difficulties of providing adequate care with limited training and resources. Nurses observed that in many families, hospitals are seen only as a last resort, as their cultural values emphasize the tremendous respect that home care provides. This preference, however, can occasionally result in inadequate care. One nurse noted, *“Some families see hospitals as a last resort because they believe home care is more respectful*,* even if it’s not always effective…like “overuse” of traditional medicine”*.

Many mistakenly believe home care is always ideal, overlooking potential medical resources and expertise shortages. While cultural norms shape preferences for care, they can come in conflict with modern healthcare, especially when traditional views on illness and death, along with misconceptions about palliative care, hinder treatment decisions. Thus, the challenge is to deliver the most effective and holistic care while being mindful of cultural values.

This study could illustrate how traditional practices build community support while highlighting the urgent need to combine them with modern medicine for better, more sustainable healthcare. Participants emphasized how traditional beliefs shape decision-making at the end of life. Cultural expectations often dictate caregiving roles, especially for women, and influence the acceptance or rejection of hospital care. While these norms provide emotional coherence to families, they sometimes clash with professional care protocols. *“In our tradition*,* dying at home is better…We have our rituals…home… It’s […] where the ancestors wait*,*”* stated a family caregiver.

The tension between modern clinical settings and ancestral practices highlights the need for cultural negotiation in care planning.

### Patient dignity

Although hospitals offer pain and symptom management, their institutional approach can compromise patient dignity, particularly when cultural practices are not considered. According to a nurse, *“It is difficult to make patients feel at home in the hospital*,* especially … when they’re used to being surrounded by family and familiar things.”* Meanwhile, as per a family caregiver, *“I wanted my mother to stay at home because in the hospital*,* she seemed like just another patient*,* … hum.not the strong woman she was.”* Another family caregiver stated, *“It’s difficult to make patients feel at home in the hospital*,* especially when they’re used to being surrounded by family and familiar things.”*

These responses highlight the difficulty of balancing effective medical care with patients’ emotional and cultural needs. Furthermore, caregivers noted that the impersonal nature of hospitals diminishes patients’ identities, reducing them to medical cases rather than individuals deserving of respect and recognition.

The emotional cost of seeing a loved one in a place so detached from their life and what they left behind is evident in this statement. The contrast reveals a gap between the hospital environment and cultural norms prioritizing family participation and individual patient needs. Further, this statement reveals a conflict between hospitals’ medical benefits and patients’ emotional and cultural well-being, suggesting a more humane, culturally aware approach is necessary to uphold patient dignity.

Caregivers often highlight the dignity of home care, emphasizing the comfort of familiar surroundings and family presence. Effective symptom management is sometimes challenging, which can potentially affect care quality. Many respondents stressed home care’s emotional and cultural benefits, especially in preserving patient dignity. Caregivers stressed that the comfort of home and family support fostered a sense of dignity and emotional well-being in their relatives. A family caregiver mentioned, *“At home*,* my grandmother was surrounded by love*,* […] which gave her dignity …*,* she died […] peacefully but we struggled to keep her comfortable due to lack of resources.”*

The importance of family presence and emotional support in preserving dignity is evident, yet limited medical resources pose a significant obstacle to effective symptom management. Nurses also appreciated the respectful atmosphere of home care, noting that patients felt more valued and cared for at home. They acknowledged significant difficulties, especially in managing symptoms, which can affect the quality of care. For instance, a nurse mentioned, *“Patients feel respected when […] they are cared for at home*,* but the inability to manage symptoms … hum … properly sometimes compromises their dignity.”*

The emotional benefits of home care contrast with its practical limitations in managing the complex medical needs of patients nearing the end of life. Although home-based care can improve patient dignity by offering emotional support and culturally sensitive care, insufficient resources and medical expertise can compromise care quality, negatively affecting patient comfort and well-being. Moreover, home-based care allowed patients greater autonomy and a sense of control in their final days. However, the lack of medication and professional support limited their physical comfort. According to a family caregiver, *“No morphine. Only prayers and what I can buy from the chemist. hum…But he screams in pain.”* Thus, hospital settings, while medically equipped, were often experienced as emotionally sterile or spiritually isolating.

### Hybrid care models

The results may imply insufficient care for palliative patients in Cameroon, regardless of whether it is hospital-based or home-based. Thus, a hybrid approach could be a viable solution, merging hospital technology with the sensitive and supportive environment of home-based care.

*If we could combine the hospital’s resources with the comfort of home*,* it […] would be the best of both worlds for patients*,*”* stated a nurse, while a family caregiver noted, *“A system where nurses regularly visit homes to provide care would help us maintain cultural traditions while ensuring better symptom management.*

The emotional support provided by home care contrasts with its limitations in handling the complex medical requirements of patients nearing the end of life. While home care can boost patient dignity with emotional and culturally appropriate support, inadequate resources and medical knowledge can hinder its success, potentially harming patient well-being.

This research illuminates the interplay of care quality, cultural norms, and patient dignity within palliative care in Cameroon and similar contexts, offering valuable insights for service improvement. Table 1 presents a comparative analysis of each care model’s benefits and challenges within Cameroon’s cultural and healthcare settings. Participants envisioned care models that combined the emotional and spiritual richness of home care with the clinical safety of hospital care; however, most acknowledged significant barriers to implementing such models, including lack of funding, infrastructure, and trained interdisciplinary teams.

**Table 1:** Comparative analysis of palliative care models in cameroon: Hospital-Based, Home-Based, and hybrid approaches

Aspect	Hospital-based care	Home-based care	Hybrid care models (Bridge)	Challenges
Cultural considerations	Care may not align with traditional beliefs about illness and death Limited family involvement due to hospital policies Language barriers in urban hospitals Cultural and spiritual dimensions of care	Respects traditional beliefs and practices Involves family and spiritual leaders Greater acceptance of cultural rituals Cultural and spiritual dimensions of care	Incorporates hospital expertise while respecting cultural norms Mobile palliative care teams ensure culturally sensitive care Encourages collaboration between hospitals and traditional healers Cultural and spiritual dimensions of care	Hospitals may not be flexible in accommodating cultural needs Home-based rituals may conflict with medical guidelines Lack of standardized cultural training for healthcare providers
Emotional support	Professional counseling available but underutilized Limited time for deep emotional engagement Some patients feel isolated in clinical environments Moral distress among nurses	Strong family support enhances emotional well-being Absence of professional mental health care Emotional burden on caregivers	Home visits by trained professionals provide emotional and psychological support Community-based care teams offer a mix of professional and familial support Digital mental health resources (if available) bridge gaps	Shortage of trained mental health professionals Stigma around psychological support Unequal access to hybrid care in rural areas Systemic barriers and resource constraints
Patient dignity	Symptom management is medically controlled The clinical setting may feel impersonal Patients may feel like a burden due to hospital bureaucracy	Greater autonomy in daily decisions Familiar surroundings enhance comfort and dignity Limited symptom control and hygiene resources	Mobile care teams ensure proper symptom management at home Flexible hospice models allow a balance of medical and personal care Family is trained to maintain patient dignity while ensuring proper care	Limited funding for hybrid care programs Insufficient integration between hospital and home care Logistical barriers in reaching remote patients

Table 1 compares hospital-based, home-based, and hybrid (or bridge) care models across the key dimensions of cultural considerations, emotional support, and patient dignity while also outlining associated challenges. Hospital care may not align with traditional beliefs and limits family involvement, while home care respects cultural rituals and involves family and spiritual leaders. Hybrid models aim to balance medical expertise with cultural norms but face challenges like hospital inflexibility and a lack of standardized training, suggesting that the staff may not be uniformly equipped to handle diverse cultural practices and beliefs.

Hospitals offer professional counseling but lack deep engagement, whereas home care provides strong family support but places emotional strain on caregivers. Meanwhile, hybrid models, despite their best efforts, struggle with mental health professional shortages and stigma—a challenge that requires our understanding and support.

Moreover, hospitals provide symptom management but may feel impersonal, home care offers autonomy but lacks medical resources, and hybrid care ensures symptom control while maintaining dignity, a factor that should reassure and instill confidence in the audience. However, funding, integration, and logistical barriers hinder hybrid models.

Evidently, each model has strengths and weaknesses, with hybrid care attempting to bridge gaps but facing systemic and logistical challenges.

## Discussion

### Hospital- and home-based care: strengths and limitations

Participants perceived hospital-based care as providing superior medical treatment that was, nevertheless, often compromised by systemic challenges hindering personalized and emotional care, whereas home-based care was valued for emotional support and cultural relevance but lacked adequate medical resources.

This study confirms the findings of existing literature that acknowledge the medical advantages of hospital-based palliative care, particularly for complex conditions [20; Hoerger et al. 25; 34]. In particular, our findings align with those highlighting how heavy workloads and time constraints in hospital settings impede the development of meaningful patient relationships and personalized care [5; 16]. The emotional detachment reported by both nurses and family caregivers in hospitals constitutes a significant limitation, especially given the crucial role psychosocial and spiritual support plays in end-of-life care [27; 49]. This is particularly salient in the Cameroonian context, where cultural and familial engagement is highly valued [9; 51].

Conversely, our study supports the perception that home-based care addresses emotional needs and cultural values better, fostering dignity and peace through familiar environments and the presence of loved ones [22; 35]. However, the study also reinforces the documented limitations of home-based care concerning medical support, with family caregivers often lacking training and resources for effective symptom management [5; 11; 44]. This imbalance highlights the necessity of integrated models that combine hospital-level clinical support with the cultural sensitivity of home care [15; 30].

### Cultural adaptation in hospital-based care

Significant tension exists between hospital protocols and the cultural practices essential for dignified end-of-life care in Cameroon. Our findings underscore the frequent failure of medically proficient hospitals to accommodate traditional rituals valued by Cameroonian families during end-of-life care, a concern also noted in other studies [5; 43]. The frustration expressed by family caregivers concerning a lack of cultural awareness in hospitals aligns with research suggesting that such disconnects can lead to emotional distress and eroded trust [3; 46]. This study reinforces the urgent need for culturally adapted care in hospital settings, emphasizing that the omission of cultural elements can compromise holistic well-being, a point supported by Agbodjavou et al. [4] [4].

The central role of spirituality, family involvement, and cultural rituals in shaping perceptions of illness and care, as highlighted in our study, is consistent with previous research in sub-Saharan African contexts [32; 41]. Specifically, our findings emphasize the importance caregivers place on honoring religious practices and maintaining traditional end-of-life rituals for preserving patient dignity and community cohesion.

### Cultural strengths and strains in home-based care

Home-based care aligns with cultural expectations and moral obligations but places a substantial burden on family caregivers, thus necessitating external support. This study confirms that home-based palliative care is deeply rooted in Cameroonian cultural expectations, aligning with findings that describe family caregiving as a meaningful act affirming social responsibility [2; [4]. While performing rituals at home strengthens emotional and spiritual dimensions of care, our research highlights the significant physical and emotional exhaustion experienced by family caregivers, often without adequate professional support concern echoed in other studies [31].

Furthermore, our findings support the argument that home-based care cannot solely rely on family dedication [42] and underscore the need for community-based support networks [10], institutional interventions like caregiver training [24], access to mobile health services, and policy changes to provide necessary resources [57]. These are crucial for transforming home-based care into a comprehensive and sustainable model.

### The impact of cultural norms and traditions on palliative care

Cultural values significantly influence perceptions of illness, caregiving, and death, creating both opportunities and challenges for palliative care delivery. Our study emphasizes how cultural norms shape the preference for home-based care as an expression of love and duty [53]. However, it also notes the potential for these ideals to obscure the heavy burden on unsupported caregivers and potentially delay seeking professional help. The reluctance to embrace hospital-based care in some contexts, due not to access issues but cultural symbolism, further complicates care delivery.

Our findings align with those of previous studies indicating that misconceptions about palliative care and cultural taboos surrounding illness and death can hinder open dialogue and delay crucial interventions [7; 53]. Thus, this study reinforces the need to perceive culture not as an obstacle but as a dynamic context within which care must be delivered, thereby necessitating strategies such as community education, culturally sensitive training, and flexible care models.

### Patient dignity in hospital-based care

While hospitals offer medical management, their institutional approach can inadvertently compromise patient dignity by overlooking emotional and cultural needs. This study supports the notion that hospital-based settings, despite providing advanced medical treatment, often struggle to maintain patient dignity due to a depersonalized environment [17; 48]. The discomfort expressed by families regarding the perceived dilution of their loved ones’ identities in hospitals aligns with the understanding that medical excellence alone does not equate to dignified care, particularly in palliative settings.

Furthermore, our findings also underscore the emotional and spiritual comfort associated with home-based care and its contribution to patient dignity through familiar surroundings and family support [37; 38]. However, the study also highlights the potential compromise to dignity in home care due to limited access to medical interventions. Thus, to protect dignity in hospitals, our findings suggest the importance of personalized care planning, humanized spaces, empathetic communication, and cultural inclusion.

### Balancing medical efficiency and emotional needs

Effective palliative care requires a balance between medical efficiency and honoring patients’ emotional and cultural realities, necessitating integrated solutions.

This study reinforces the understanding that palliative care extends beyond symptom management to include patients’ emotional and cultural well-being [10]. While hospitals excel in treating disease, their focus on efficiency can inadvertently sideline emotional and cultural needs. Conversely, while home care preserves emotional comfort, it may lack necessary clinical support. Thus, our findings advocate for integrated solutions such as resource support for home care, holistic models in hospitals, and flexible hybrid care systems [21; 56].

### Hybrid care models: bridging the gap in palliative care

Hybrid care models, integrating hospital expertise with community-based home care, offer a promising solution to address the dual challenges of limited infrastructure and the need for culturally sensitive care in Cameroon.

Our study supports the growing understanding that neither hospital-based nor home-based care alone fully meets the complex needs of end-of-life patients in resource-limited settings like Cameroon, thus highlighting the potential of hybrid models [55]. The tension between clinical efficacy and cultural appropriateness in the current landscape points to the need for such integrated approaches [6; 26]. The aspirations voiced by both nurses and family caregivers for a system that combines hospital resources with home comfort further validate this direction.

Drawing from international experiences, our findings emphasize the importance of community-based palliative care teams, telemedicine integration, resource support for home-based care, and culturally sensitive training for providers in developing an effective hybrid system tailored to the Cameroonian context [1; 8; 13]. The potential benefits of such models include improved equity, sustainability, and patient satisfaction [33; 47].

### Designing a hybrid model for palliative care in Cameroon

An effective palliative care system in Cameroon must integrate clinical efficiency with cultural relevance through a hybrid model built on community-based teams, telemedicine, resource support, and culturally sensitive training.

The study not only highlights several key areas for future exploration but also paves the way for groundbreaking research. First, it calls for further investigation into the feasibility of hybrid care approaches that integrate hospital-based services with culturally grounded home care practices. Additionally, there is a need to develop and assess training programs tailored to local cultural contexts, ensuring that healthcare providers are equipped to deliver sensitive and effective palliative care. The research also underscores the importance of designing scalable, community-based pain management interventions that can operate effectively in low-resource settings. Ultimately, the study emphasizes the importance of incorporating patient perspectives in care planning to ensure that models of care accurately reflect patients’ needs, values, and preferences.

Our research supports the evidence that a hybrid model can improve symptom control, access to services, and preserve patient dignity [19; 58; 52]. By investing in this integrated approach, Cameroon can establish a palliative care system that is both medically effective and culturally compassionate, offering a valuable model for similar contexts worldwide.

## Study limitations and future research

This study offers valuable insights into the experiences and perceptions of nurses and family caregivers involved in palliative care in Cameroon, contributing meaningfully to an under-researched area. Nonetheless, several limitations should be acknowledged to contextualize its findings.

First, the study was geographically limited to specific regions, which, while allowing for in-depth exploration, may not capture the full diversity of perspectives across Cameroon, as variations in healthcare infrastructure, cultural practices, and socioeconomic conditions in other areas may shape different experiences of palliative care delivery.

Second, the sample size, although sufficient for qualitative inquiry, was relatively narrow and excluded key stakeholders such as patients, policymakers, and community leaders. The absence of patient voices is particularly notable, as their perspectives are crucial for understanding how care models affect autonomy, dignity, and comfort. Including a broader range of participants would have enriched the analysis and offered a more comprehensive view of systemic challenges.

In addition, the study’s reliance on qualitative, perception-based data limits its ability to assess care outcomes or quality in objective terms. While personal narratives are essential for understanding lived experiences, future research should integrate quantitative methods to support and extend these findings.

The strong emphasis on cultural and spiritual dimensions, central to care in this context, may also introduce a degree of cultural specificity that limits the generalizability of findings both within and beyond Cameroon. Furthermore, although the proposed hybrid care model represents a promising avenue, the study did not fully examine its feasibility, including practical considerations such as funding, staffing, and infrastructure needs.

Finally, while resource constraints were acknowledged, the study did not delve into the structural drivers behind these limitations. Therefore, a deeper exploration of issues like healthcare financing, access to essential medications, workforce shortages, and gaps in palliative care training is needed to inform effective policy responses.

Despite these limitations, this study provides a valuable foundation for future research. Expanding the geographic and participant scope, incorporating mixed methods, and investigating the implementation of hybrid care models are key next steps toward improving palliative care in resource-constrained, culturally diverse settings.

## Strengths of the study

This study offers a unique exploration of hospital- and home-based palliative care in Cameroon, using a qualitative descriptive design with an interpretive lens and thematic analysis of semi-structured interviews. It addressed a critical gap in global health literature, particularly the lack of qualitative research on the lived experiences of nurses and family caregivers in Cameroonian palliative care. In a context marked by limited resources and strong cultural traditions, the study provides valuable insights into care quality, cultural adaptation (e.g., integrating familial caregiving with formal medical support), and patient dignity.

A key strength lies in its cultural sensitivity. The study thoughtfully explored how traditions, beliefs, and community values shape care preferences and practices, offering culturally relevant guidance for developing respectful, effective care models. Rigor is enhanced through strategies that ensure trustworthiness, such as data triangulation and reflexivity.

The study’s focus on patient dignity is particularly notable. By examining how different care environments affect emotional well-being and respect, the study positions dignity as a central indicator of quality palliative care.

Rich qualitative data, including direct participant quotes, adds authenticity and depth. These narratives reveal the nuanced realities of caregivers’ experiences—details that are often missed in quantitative studies. The research also outlined systemic challenges, including staff shortages, high caseloads, and logistical barriers, underscoring the need for context-specific solutions.

Significantly, the study proposes a hybrid care model that combines medical expertise with culturally grounded, community-based support. Though further research is needed to assess its feasibility, this model offers a promising direction for care delivery in resource-constrained settings.

The study contributes to global palliative care discourse by illuminating how cultural context, systemic limitations, and patient-centered values intersect, offering transferable insights for other LMICs.

## Recommendations

Enhancing palliative care in Cameroon: key recommendations that are vital for progress.

Palliative care in Cameroon demands a comprehensive, culturally sensitive approach that respects and values the diverse cultural practices and beliefs surrounding illness and death. This research has led to several key recommendations, including the development of policies that integrate cultural practices, training and support for healthcare professionals, community engagement and awareness, resource allocation, and the establishment of robust referral mechanisms between hospital and home-based care. These insights have been compiled into a policy brief for the Department of Health in Cameroon, aiming to enhance inpatient and home-based palliative care, ensuring it is culturally sensitive, effective, and supportive of patients and their families.

1. Strengthening healthcare professional capacity and cultural competence
  Investing in education and well-being of healthcare professionals is vital for improving palliative care delivery across all settings.
  *Comprehensive Palliative Care Training*: Offers specialized training for all healthcare professionals, including nurses, with a focus on symptom management, effective and practical communication skills, and supporting families throughout the decision-making process.
  *Ongoing Cultural Competence and Sensitivity Training*: A Continuous Effort to integrate cultural sensitivity and competence training into both nursing education and continuous professional development. This should enhance understanding of diverse cultural practices, beliefs surrounding illness and death, and the emotional needs of patients and families.
  *Emotional and Psychological Support for Staff*: Implement robust emotional support systems for nurses and other healthcare providers, such as counseling services and peer support groups, to help them manage the psychological challenges inherent in palliative care.
  *Adequate Staffing and Resources*: Ensure appropriate nurse-to-patient ratios in hospitals and provide sufficient resources and funding to support palliative care services, preventing caregiver burnout and enabling quality care.

2. Empowering and supporting family caregivers in Home-Based care
  Strengthening the capacity of family caregivers is crucial for the success and sustainability of home-based palliative care.
  *Caregiver Training Programs*: Develop accessible training programs for family caregivers to equip them with practical skills for symptom management (including pain relief), basic medical care, and emotional support, while respecting traditional practices.
  *Improved Access to Home Care Resources*: Ensure that family caregivers have reliable access to essential medical resources, such as medications, equipment, and professional guidance (e.g., through mobile health units or telehealth services), to enhance the quality and safety of home care.
  *Community-Based Support Networks*: Foster collaboration with local community organizations and leaders to establish robust networks that provide social, emotional, and practical support for families undertaking home-based care, aligning with community traditions and values.

3. Fostering culturally integrated and Patient-Centered care models
  Healthcare systems must actively integrate cultural practices and patient preferences to ensure dignified and holistic palliative care.
  Flexible Protocols for Cultural Practices: Adapt hospital policies and protocols to explicitly allow and accommodate essential cultural and spiritual rituals during critical moments, such as designated prayer spaces or family-specific practices.
  Patient and Family Involvement in Care Planning: Systematically involve patients (where appropriate) and their families in all care decisions, ensuring that medical interventions align with their cultural preferences, values, and expressed needs.
  Collaboration with Traditional Healers: Explore and foster partnerships between modern healthcare systems and traditional healers to incorporate culturally appropriate and respectful practices into palliative care plans where beneficial.

4. Establishing seamless referral pathways and hybrid care solutions
  Ensuring a seamless transition between hospital and home care is a critical aspect of palliative care. This requires the development of integrated systems that guarantee continuity and accessibility, providing reassurance to patients and their families about the quality of care they will receive.
  *Robust Referral Mechanisms*: Develop clear, standardized referral pathways and communication protocols to facilitate seamless transitions between hospital-based and home-based palliative care, ensuring patients receive appropriate care at all stages.
  *Implementation of Hybrid Care Models*: Develop and pilot hybrid care models that strategically blend the medical expertise of hospital settings with the comfort and cultural relevance of home care. This could include regular home visits by healthcare professionals, scheduled hospital visits for complex needs, and expanded use of telemedicine for remote support and guidance, particularly in rural areas.

5. Policy advocacy and public awareness
  Creating an enabling environment for palliative care necessitates systemic change and a deeper understanding of the public.
  *Development of National Palliative Care Policies*: Advocate for government-led national policies that formally recognize, support, and regulate palliative care services, including ensuring consistent access to essential palliative care medications.
  *Launching widespread awareness campaigns* is crucial to educate the general population about the benefits of palliative care, clarify misconceptions, and reduce stigma associated with serious illness and death. This will make the audience feel more informed and educated about palliative care, thereby fostering a more supportive environment for patients and their families.
  *Integration into Primary Healthcare*: Incorporate basic palliative care principles and services into primary healthcare to ensure more comprehensive, accessible, and early intervention for patients and families.
  *Regular Service Evaluation*: Establish mechanisms for the periodic evaluation and monitoring of palliative care services across Cameroon to identify gaps, track progress, and continuously inform improvements.

## Conclusion

This study explored the nuanced perceptions of nurses and family caregivers in Cameroon regarding the quality, cultural relevance, and impact on patient dignity within hospital versus traditional home-based palliative care settings. Our findings reveal that patient and family decisions about palliative care are profoundly complex, shaped by a dynamic interplay of practical considerations, cultural values, and emotional needs.

Hospital care consistently demonstrated advantages in symptom management and access to medical expertise, underscoring the crucial role of medical knowledge in palliative care. However, the rigid nature of hospital systems often inadvertently compromises patient dignity and fails to fully account for vital cultural values, such as the crucial role of family involvement and emotional well-being. Conversely, home-based care was highly valued for its cultural sensitivity, providing a familiar and supportive environment that significantly enhanced emotional well-being and upheld patient dignity. Nevertheless, the inherent limitations in resources and medical knowledge at home frequently impacted the quality of medical care, particularly in the effective management of symptoms.

The research also shed light on the significant difficulties encountered by nurses in both environments. In hospitals, high patient volumes and inflexible systems presented challenges in delivering care that was both culturally appropriate and individually tailored. Nurses providing home care faced considerable logistical and resource constraints, limiting their ability to provide comprehensive support. Additionally, family caregivers, who play a crucial role in the palliative care process, often struggle to balance traditional expectations with the practical demands of caregiving, frequently with insufficient external support.

Ultimately, this study demonstrates that the multifaceted needs of palliative care patients in Cameroon are not fully met by either hospital-based or traditional home-based care in isolation. The urgent need for a promising healthcare solution lies in developing hybrid models that seamlessly blend the medical expertise available in hospitals with the emotional support and cultural understanding inherent in home care. By leveraging community-based care teams, integrating telemedicine, and implementing culturally sensitive training for providers, comprehensive, patient-centered care can be achieved.

In conclusion, effective palliative care strategies for Cameroon must be both culturally appropriate and resource-responsive. By thoughtfully considering both the medical imperatives and deep-seated cultural aspects of care, policymakers and healthcare providers can significantly improve patient and family well-being while respecting their rich cultural heritage. Future research should prioritize piloting and rigorously assessing these hybrid care models to determine their effectiveness, practicality, and adaptability across diverse cultural contexts and resource-constrained environments.

## Data Availability

No datasets were generated or analysed during the current study.
